# Sterile alpha and TIR motif-containing protein 1 is a negative regulator in the anti-bacterial immune responses in nile tilapia (*Oreochromis niloticus*)

**DOI:** 10.3389/fimmu.2022.940877

**Published:** 2022-07-19

**Authors:** Nguyen Bao Trung, Tan-Phat Nguyen, Hao-Yun Hsueh, Jiun-Yan Loh, Eakapol Wangkahart, Alice Sui Fung Wong, Po-Tsang Lee

**Affiliations:** ^1^ Department of Aquaculture, National Taiwan Ocean University, Keelung, Taiwan; ^2^ College of Aquaculture and Fisheries, Can Tho University, Can Tho, Vietnam; ^3^ Centre of Research for Advanced Aquaculture (CORAA), UCSI University, Kuala Lumpur, Malaysia; ^4^ Laboratory of Fish Immunology and Nutrigenomics, Applied Animal and Aquatic Sciences Research Unit, Division of Fisheries, Faculty of Technology, Mahasarakham University, Khamriang Sub-District, Mahasarakham, Thailand

**Keywords:** *Aeromonas*, inflammation, innate immunity, SARM1, *Streptococcus*, TLR, negative regulator, NFκB

## Abstract

Nile tilapia (*Oreochromis niloticus*) is one of the most important food fish in the world. However, the farming industry has encountered significant challenges, such as pathogen infections. Toll-like receptors (TLRs) play an essential role in the initiation of the innate immune system against pathogens. Sterile alpha and TIR motif-containing protein 1 (SARM1) is one of the most evolutionarily conserved TLR adaptors, and its orthologs are present in various species from worms to humans. SARM1 plays an important role in negatively regulating TIR domain-containing adaptor proteins inducing IFNβ (TRIF)-dependent TLR signaling in mammals, but its immune function remains poorly understood in fish. In this study, *O. niloticus* SARM1 (*OnSARM1*) was cloned and its evolutionary status was verified using bioinformatic analyses. mRNA expression of *OnSARM1* was found at a higher level in the trunk kidney and muscle in healthy fish. The examination of its subcellular location showed that the OnSARM1 was detected only in the cytoplasm of THK cells, and colocalized with OnMyD88, OnTRIF and OnTRIF in small speckle-like condensed granules. The transcript levels of *OnMyD88*, *OnTIRAP*, *OnTRIF*, and downstream effectors, including *interleukin* (*IL*)-*1β*, *IL-8*, *IL-12b* and type I *interferon* (*IFN*)*d2.13*, were regulated conversely to the expression of *OnSARM1* in the head kidney from *Aeromonas hydrophila* and *Streptococcus agalactiae* infected fish. Moreover, the treatment of THK cells with lysates from *A. hydrophila* and *S. agalactiae* enhanced the activity of the NF-κB promoter, but the effects were inhibited in the OnSARM1 overexpressed THK cells. Overexpression of OnSARM1 alone did not activate the NF-κB-luciferase reporter, but it suppressed OnMyD88- and OnTIRAP-mediated NF-κB promoter activity. Additionally, OnSARM1 inhibited the mRNA expression of proinflammatory cytokines and hepcidin in *A. hydrophila* lysate stimulated THK cells. Taken together, these findings suggest that OnSARM1 serves as a negative regulator by inhibiting NF-κB activity, thereby influencing the transcript level of proinflammatory cytokines and antimicrobial peptides in the antibacterial responses.

## Introduction

Aquaculture is a well-known activity that has spread globally. In 2020, the Food and Agriculture Organization (FAO) released statistical data showing that between 2001 and 2018, the global aquaculture production of farmed aquatic animals increased at a rate of 5.3% per year (1). Aquaculture will continue to be a leading source of global fish production, continuing a decades-long trend. The production of aquaculture is expected to reach 109 million tonnes in 2030, increasing by 32% (26 million tons) from 2018 (1).

Globally, tilapia is the second most important farmed fish, after carp. Despite the impact of COVID-19, the worldwide production of farmed tilapia increased by 3.3% in 2020, surpassing 6 million tons for the first time (2). Tilapia is a fast-growing fish that is relatively disease-resistant, yet several pathogenic microorganisms have jeopardized its production (3). The farming industry has encountered significant challenges from infectious pathogens, such as *Streptococcus agalactiae*, *Streptococcus iniae*, *Aeromonas hydrophila*, *Edwardsiella tarda*, and *Flavobacterium* spp. (4–7). Furthermore, the tilapia lake virus (TiLV) is a tilapia virus that causes mass deaths (8).

Innate immunity is an essential and widely distributed form of immunity representing the first line of defense against pathogens (9). Pathogens are identified by a variety of pattern-recognition receptors (PRRs) in the innate immune system, including toll-like receptors (TLRs), retinoic acid-inducible gene (RIG)-I-like receptors, the nucleotide-binding oligomerization domain (NOD)-like receptors, and C-type lectin receptors (10). The TLR family is one of the most important families in the PRRs, which recognize pathogens extracellularly or in intracellular organelles, such as endosomes and lysosomes (11).

Five TLR adaptor proteins have been recognized: the myeloid differentiation factor 88 (MyD88), the TIR domain-containing adaptor protein (TIRAP), the TIR domain-containing adaptor protein inducing IFNβ (TRIF), the TRIF-related adaptor molecules (TRAM), and the Sterile alpha and TIR motif-containing protein 1 (SARM1) (12). SARM1, the latest identified TLR adaptor protein (13), has been shown to be a negative regulator of TRIF in humans, in contrast to the other four adaptors that have shown activating effects (14). SARM1 is a highly conserved protein present in a variety of organisms, including the horseshoe crab (*Limulus polyphemus)*, roundworm (*Caenorhabditis elegans*), amphioxus (*Branchiostoma belcheri tsingtauense*), zebrafish (*Danio rerio*), grass carp (*Ctenopharyngodon idella*), and whiteleg shrimp (*Litopenaeus vannamei*), revealing that it is ancient in its origins (13, 15–19).

SARM1 has been cloned from mammals and a few fish species and studies have uncovered its role as a negative regulator in anti-viral pathways (13, 14, 20), yet its functions in anti-bacterial responses have never been properly scrutinized. To investigate this, we performed *in vitro* and *in vivo* bacterial stimulation/infection models and report that OnSARM1 negatively regulates NF-κB activity, thereby influencing the transcript level of proinflammatory cytokines and antimicrobial peptides.

## Materials and methods

### Experimental fish, infection, and sample collection

The Nile tilapia (*Oreochromis niloticus*) NT1 strain was generously provided by Prof. Hong-Yi Gong (Department of Aquaculture, National Taiwan Ocean University). The procedures for sampling tissues from healthy fish are described by Trung and Lee (21).

A total of 80 fish (average weight 45.8 ± 6.8 g) were acclimatized in four 90 L glass tanks (n = 20 each) that were aerated and supplied with dechlorinated tap water (28 ± 2°C). The photoperiod for each tank was maintained at 12 h light/12 h dark. *A. hydrophila* and *Streptococcus agalactiae*, which had been isolated from diseased tilapia, were cultured on tryptic soy agar (TSA, Difco) at 28°C overnight. Cells were washed off with sterile phosphate-buffered saline (PBS, pH 7.4) and adjusted to the desired concentration. The experimental fish were weighed and injected intraperitoneally with PBS (control group), *A. hydrophila* (4 × 10^5^ colony forming units (CFU)/g of fish body weight), or *S. agalactiae* (1.3 × 10^4^ CFU/g of fish body weight) (n = 20 per group) (21). Tissues were collected (five fish per group) at 4, 8, 24, and 72 h post injection (hpi) and preserved in RNA*later* solution (Invitrogen, CA, US). Fish handling and experiments were conducted following the guidelines of the Institutional Animal Care and Use Committee, which is approved by National Taiwan Ocean University (code: 109042).

### Total RNA extraction, cDNA synthesis, and quantitative real-time polymerase chain reaction (qRT-PCR) assay

Total RNA was extracted using a TRI Reagent (Sigma-Aldrich, US) according to the manufacturers’ instructions. RNA quantity and purity were determined using a SpectraMax QuickDrop Spectrophotometer (Molecular Devices, US). Reverse transcription was conducted with 5 μg of total RNA using RevertAid™ reverse transcriptase (200 U/µl, Thermo Fisher Scientific, US). cDNA was diluted with 40 μl of RNase-free distilled water for gene cloning and stored at -20°C (21). The iScript™ gDNA Clear cDNA Synthesis Kit (BioRad, US) was used for cDNA synthesis for qRT-PCR analysis. The *elongation factor-1α* (*EF-1α*) (a reference gene) was used for normalization. Gene expression of *OnSARM1*, *OnMyD88*, *OnTIRAP*, *OnTRIF*, *interleukin* (*IL*)*-1β*, *IL-8*, *IL-12a*, *IL-12b*, *tilapia piscidin* (*TP*) *2, TP3, TP4, hepicidine, tumor necrosis factor* (*TNF*)*-α*, *major histocompatibility complex class Ia* (*MHC Ia*), and type I *interferon* (*IFN*)*d2.8* and *IFN2.13* were measured using a StepOnePlus Real-Time PCR instrument (Thermo Fisher Scientific) using the primers listed in **Supplementary Table 1**. The qRT-PCR reaction mixture (20 μl) was composed of 4 μl of cDNA template, 2 μl of specific primers, 4 μl of PCR-grade water, and 10 μl of RealQ Plus 2 × Master Mix Green (Ampliqon, Denmark). Reaction mixtures were incubated for 10 min at 95°C, followed by 40 cycles of 15 s at 95°C, and finally 1 min at 60°C. A melting curve was generated by increasing the temperature from 60°C to 95°C with 0.3°C increments.

### Cloning of *OnSARM1* and plasmid constructions

To clone Nile tilapia *SARM1*, a set of primers was designed based on the complete coding region of the *OnSARM1* sequence (**Supplementary Table 1**). The PCR reactions were conducted using MyTaq Red Mix 2× (Bioline, UK). The PCR thermal cycling was performed *via* an initial denaturation at 95°C for 1 min, followed by 35 cycles at 95°C for 15 s, 53°C for 30 s, and 72°C for 1.5 min, and a final extension at 72°C for 10 min. The obtained PCR product was cloned into the pGEM-T Easy Vector (Promega, US) and sequenced using Genomics Ltd.

The OnSARM1-GFP and OnSARM1-3×FLAG expression plasmids were prepared using primers GFP-*Nhe*I-OnSARM1-F and GFP-*Hind*III-OnSARM1-R, and FLAG-*Hind*III-OnSARM1-FL-F and FLAG-*XbaI*-OnSARM1-FL-R, respectively (**Supplementary Table 1**). Q5^®^ High-Fidelity DNA Polymerase (New England Biolabs, US) was used, and the PCR reactions were set up as follows: one cycle at 98°C for 30 s, followed by 35 cycles at 98°C for 15 s, 62°C for 30 s, 72°C for 90 s, and terminated with one cycle of 72°C for 10 min and 95°C for 60 s. The purified PCR products and empty pTurbo-GFP-N (Evrogen, Russia) or p3×FLAG-CMV-14 vector (Sigma-Aldrich) were ligated together after restriction enzyme digestion. Ligation products were transformed into ECOS™ 101 Competent Cells (DH5α) (Yeastern Biotech, Taiwan). Positive colonies were screened using vector-specific primers (**Supplementary Table 1**), and colonies with correct insert size were grown in LB broth with ampicillin (100 µg/ml) (for p3×FLAG-CMV-14 vector) or kanamycin (50 µg/ml) (for pTurboGFP-N vector) for plasmid preparation.

### Bioinformatics

Homologous sequences were searched through the BLAST algorithm (http://blast.ncbi.nlm.nih.gov/Blast.cgi). The nucleotide sequence of *OnSARM1* was translated using the ExPASy translate tool (http://web.expasy.org/translate/). The molecular weight of the protein was calculated using the Compute pI/Mw tool (http://web.expasy.org/compute_pi/). A phylogenetic tree was constructed using the neighbor-joining method in Molecular Evolution Genetics Analysis software version 7 (MEGA7), supported by 10,000 bootstrap repetitions with the Poisson model for amino acid substitution and pairwise deletion for gap treatment (22). EMBOSS Needle online software (https://www.ebi.ac.uk/Tools/psa/emboss_needle/) was used to identify the sequence identity and similarity between vertebrates OnSARM1 (23). Synteny analysis was performed *via* the Genomics v100.01 program (24). Simple Modular Architecture Research Tool (SMART) (http://smart.embl-heidelberg.de/) was used to predict the deduced amino acid sequence of *OnSARM1* (25). Multiple alignments were performed using the ClustalW program (https://www.ebi.ac.uk/Tools/msa/clustalo/).

### Cell culture

A tilapia head kidney (THK) cell line (26) was cultured at 27°C in Leibovitz medium (L-15, Gibco) containing 10% Fetal Bovine Serum (FBS, Corning) and 1% penicillin (100 U/ml)/streptomycin (100 μg/ml) (P/S, Gibco). Human embryonic kidney (HEK) 293 cells were provided by Prof. Pin-Wen Chiou (Department of Aquaculture, National Taiwan Ocean University) and were cultured in Dulbecco’s Modified Eagle’s Medium (DMEM, GE Healthcare Life Sciences) containing 10% FBS and 1% P/S at 37°C with 5% CO_2_ in a humidified incubator. When the cells reached 70-80% confluency, they were passaged or used for seeding.

### Cellular localization of OnSARM1

HEK 293 and THK cells (1 × 10^5^ cells per well) were seeded onto glass coverslips in 24-well culture plates with 1 ml culture medium 16 h before transfection. The following day, 1,000 ng of pTurbo-OnSARM1-GFP plasmids in serum-free medium was mixed with TurboFect Transfection Reagent (Thermo Fisher Scientific) and incubated for 20 min at room temperature following the manufacturer’s instructions before being added to the cells. Forty-eight hours after transfection, the cells were washed thrice with 500 μl of Hank’s Balanced Salt Solution (HBSS; Thermo Fisher Scientific) and fixed with 500 μl of 4% paraformaldehyde (PFA) at room temperature for 15 min. Subsequently, cells were washed thrice with HBSS and incubated for 30 min with HBSS containing Alexa Fluor 546 phalloidin (Thermo Fisher Scientific) in a darkened room. After cells were washed thrice with HBSS again, cells fixed on the coverslip were stained with VECTASHIELD Antifade Mounting Medium containing 4’, 6-diamidino-2-phenylindole (DAPI; Vector Laboratorie, US) on a microscope slide, and the coverslip was sealed with nail polish. Finally, the sealed coverslips were observed using a confocal microscope (Zeiss LSM 880 with Airyscan, Germany).

### Co-localization of OnSARM1 and other TLR adaptors

For the colocalization studies, THK cells (4 × 10^4^ cells per well) were seeded onto glass coverslips in 24-well culture plates, as mentioned above; this was followed by co-transfection with OnSARM1-3×FLAG and HA-tagged OnTRIF, OnTIRAP (27) or OnMyD88 (21) expression plasmids, respectively. The cells were fixed 48 h after transfection; this was followed by permeabilizing with PBS containing 1% (v/v) triton-X100 at room temperature for 5 min and blocking with PBS containing 10% bovine serum albumin (BSA) for 30 min at 37°C. Subsequently, the cells were incubated with PBS containing 3% BSA and monoclonal anti-FLAG M2 mouse antibodies (1:200, Sigma-Aldrich) or anti-HA rabbit antibodies (1:200, clone C29F4, Cell Signaling) for 2 h at 37°C. Alexa Flour™ 488 goat anti-mouse IgG (1:1000, Invitrogen) and Alexa Flour™ 568 goat Anti-rabbit IgG (1:1000, Invitrogen) were used in dark to visualize FLAG tagged (green) or HA tagged proteins (red), respectively. The cells were then stained with VECTASHIELD Antifade Mounting Medium containing DAPI to visualize the nucleus (blue). The images were captured using confocal microscopy (Zeiss LSM 880 with Airyscan). The colocalization efficiency was quantified by observing the overlap of the fluorescence intensity peaks along the white line by line scan analysis profiles (ZEISS ZEN 2.6 (blue edition) software).

### Western blotting

Western blotting was conducted according to procedures described in previous research (28). HEK 293 cells were seeded in 24-well plates at a density of 1 × 10^5^ cells per well the day before transfection. The TurboFect Transfection Reagent was used to transfect cells with the indicated plasmids or left untreated as a negative control. Plasmids encoding Nile tilapia MyD88, TRIF, and TIRAP were prepared previously (21, 27). Cell lysates from transfected cells were separated by gradient SDS-PAGE (Bionovas, Canada) before being transferred to a PVDF membrane (Millipore, US). The membrane was immunoblotted with the anti-TurboGFP (d) antibody (1:1000) (Evrogen) or ANTI-FLAG M2 antibody (1:1000) (Sigma-Aldrich). The membrane was then incubated with peroxidase-conjugated AffiniPure goat anti-rabbit or goat anti-mouse IgG (H+L) antibodies (1:5000, Jackson ImmunoResearch Laboratories, US). LuminolPen (Visual Protein) was used to mark the pre-stained molecular weight standard markers, and a SuperLight Chemiluminescent HRP Kit (Bionovas) was used for protein detection. The membrane was visualized using the UVP GelStudio PLUS Touch Imaging System (Analytik Jena, Germany).

### Luciferase assay

To investigate the luciferase activity induced by OnMyD88, OnTRIF, OnTIRAP, and OnSARM1 expressing plasmids, THK cells (5 × 10^4^ cells per well) were seeded in 48-well culture plates with 500 μl of culture medium 16 h before transfection. The cells were then transfected with 250 ng of each expression plasmid or a corresponding empty vector (pcDNA3.0-HA for OnMyD88, OnTRIF, and OnTIRAP and p3×FLAG-CMV-14 vector for OnSARM1), with 250 ng of pNF-κB-Luc (Clontech). Forty-eight hours post transfection, luciferase activity was determined using the luciferase reporter assay system (GeneCopoeia, US) according to the manufacturer’s instructions. Each experiment was performed in triplicate.

To verify the influence of OnSARM1 on the NF-κB activity mediated by OnMyD88, OnTRIF, and OnTIRAP, THK cells were transfected with 125 ng of OnMyD88, OnTRIF, or OnTIRAP plasmids with or without 125 ng of OnSARM1-3×FLAG expression plasmids, with 250 ng of pNF-κB-Luc. Luciferase activity was determined 48 h post transfection.

To study the effects of overexpressing OnSARM1 during antibacterial responses, THK cells were transfected with a pNF-κB-Luc reporter (250 ng) with empty vector or OnSARM1-3×FLAG expression plasmids (250 ng). Forty-eight hours post transfection, the cells were treated with lysates from *A. hydrophila* (10 μg protein/ml culture medium) and *S. agalactiae* (10 μg protein/ml culture medium) for 16 h, followed by luciferase assay analysis.

### Gene expression analyses in OnSARM1 overexpressed THK cells post *A. hydrophila* stimulation

THK cells (2.5 × 10^5^ cells per well in 12 well plate) were seeded the night before transfection. The next day, the cells were transfected with an empty vector (2000 ng per well) or OnSARM1-3×FLAG expression plasmids mixed with an empty vector (1000 ng each). The cells were then stimulated for 4 h with lysates from *A. hydrophila* (10 μg protein/ml culture medium) 48 h post transfection; this was followed by RNA extraction, cDNA synthesis and gene expression analysis, as described above.

### Statistical analysis

SPSS 25 software (IBM, US) was used to analyze all quantitative real-time PCR data. The expression levels of the *OnSARM1* were first normalized to the housekeeping gene *EF-1α*, and log_2_ transformed (29). An unpaired-sample t-test was performed to analyze the significant differences between the treatment and control groups in gene expression levels at each time point. One-way ANOVA (analysis of variance) and Tukey’s test were conducted to analyze the differences in each group in the luciferase assay. P-value < 0.05 was considered significantly different.

## Results

### Cloning, phylogenetic analysis, and sequence comparison

The complete coding sequence (CDS) of *OnSARM1* was cloned, and the nucleotide and deduced amino acid sequences of *OnSARM1* are shown in **Supplementary Figure 1**. The cloned *OnSARM1* cDNA sequence was 2206 bp in length, including a partial 34 bp of 5′-UTR and a partial 36 bp of 3′-UTR. The open reading frame (ORF) of *OnSARM1* was 2136 bp, encoding for 711 amino acids.

**Figure 1:**
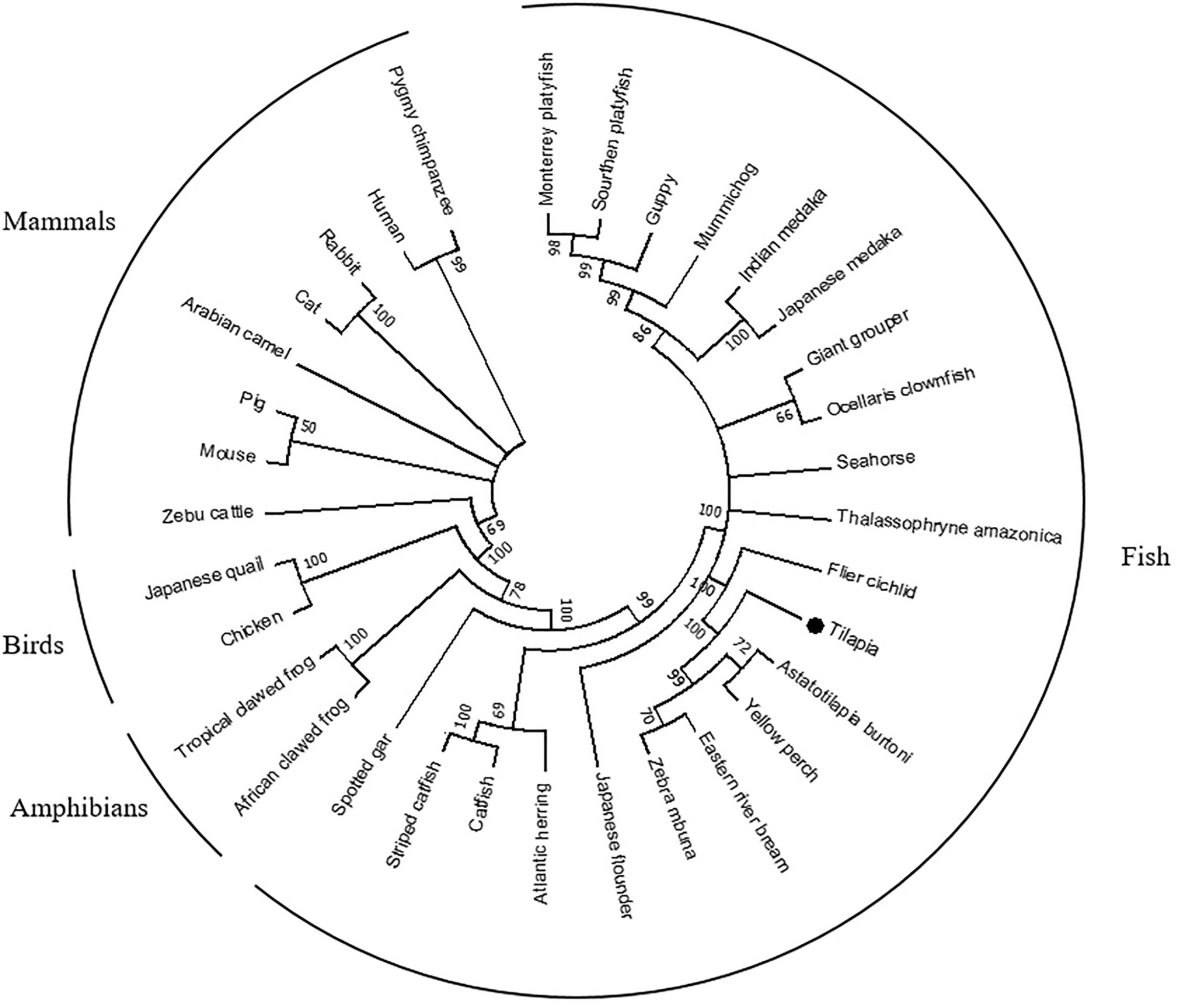
A phylogenetic tree of selected vertebrate SARM1 genes. The phylogenetic tree was constructed from multiple alignments of amino acid sequences (ClustalW), using the Maximum Likelihood method based on the JTT matrix-based model and supported by 10,000 bootstrap repetitions in the MEGA7 program. Data were analyzed by applying Neighbor-Join and BioNJ algorithms to a matrix of pairwise distances estimated using a JTT model and then selecting the topology using the superior log likelihood value. A black dot shows the Nile tilapia gene reported in this study. Accession numbers of the sequences used to construct the tree are listed in **Supplementary Table 2**.

To investigate the evolutionary relationships of *OnSARM1*, a phylogenetic tree consisting of 33 SARM1 amino acid sequences from selected vertebrate species was constructed (**Figure 1**; accession numbers are in **Supplementary Table 2**). The putative SARM1 of Nile tilapia clustered with other fishes, forming a clade separate from the clades of avian, amphibian, and mammalian sequences. OnSARM1 was closer to flier cichlid, *Astatotilapia burtoni*, yellow perch, eastern river bream, and zebra mbuna, which all fall within the Actinopterygii class.

**Figure 2:**
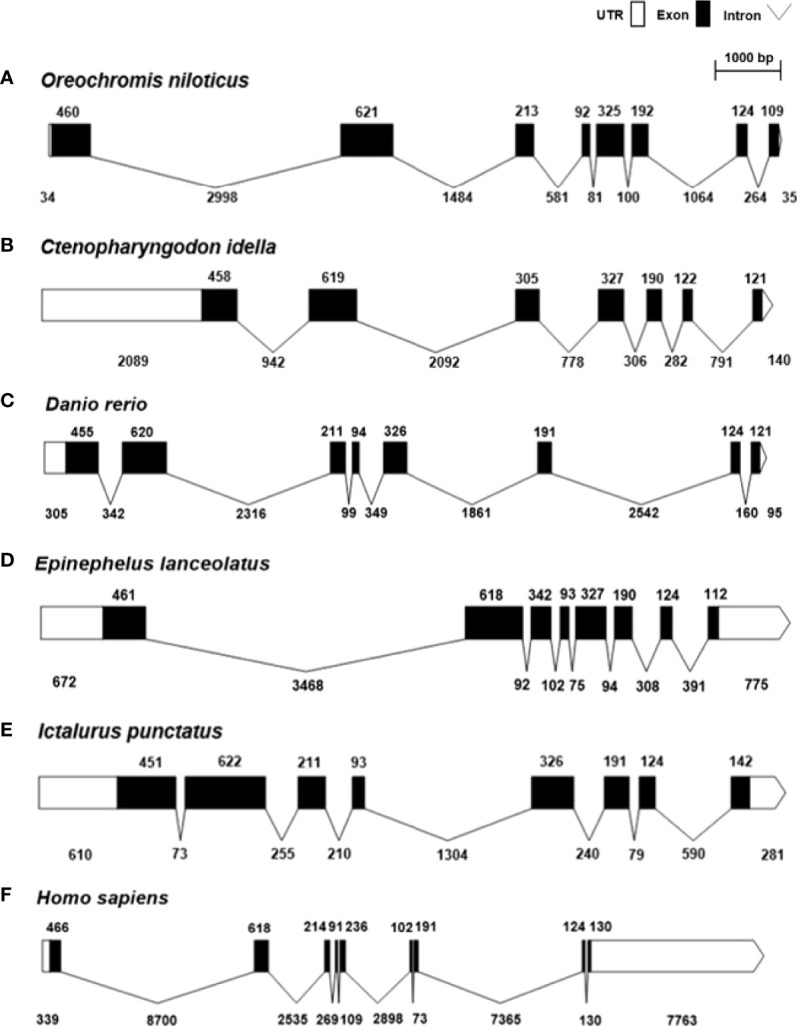
Genomic structure of SARM1 from **(A)** *Oreochromis niloticus* (XM_003458624), **(B)** *Ctenopharyngodon idella* (JX887876), **(C)** *Danio rerio* (NM_001130596), **(D)** *Epinephelus lanceolatus* (XM_033631735), **(E)** *Ictalurus punctatus* (XM_017491372), and **(F)** *Homo sapiens* (NM_015077). The white and black bars represent untranslated regions (UTRs) and exons, respectively, while introns are indicated by thin black lines.

**Figure 2** shows the genomic structure of *SARM1s* from Nile tilapia and other selected vertebrates. Genomic structure analysis revealed that except for *Ctenopharyngodon idella* and *Homo sapiens*, all of the selected teleost *SARM1* shared the same structure, which consisted of eight exons and seven introns. *Ctenopharyngodon idella SARM1*(*CiSARM1*) consisted of seven exons and six introns, while *Homo sapiens SARM1* consisted of nine exons and eight introns (**Figures 2B, F**).

Protein identities and similarities of the SARM1s were determined among selected vertebrates (**Supplementary Table 3**). The results showed that *OnSARM1* shared high homology with other vertebrate SARM1s (56.9–91.7% identity and 70.6–95.9% similarity), with the highest homology to the SARM1 from *Lates calcarifer* (91.7% identity and 95.9% similarity) and the lowest homology to the SARM1 from *Mus musculus* (56.9% identity and 70.6% similarity).

### Domain organization and genome synteny analyses

**Figure 3:**
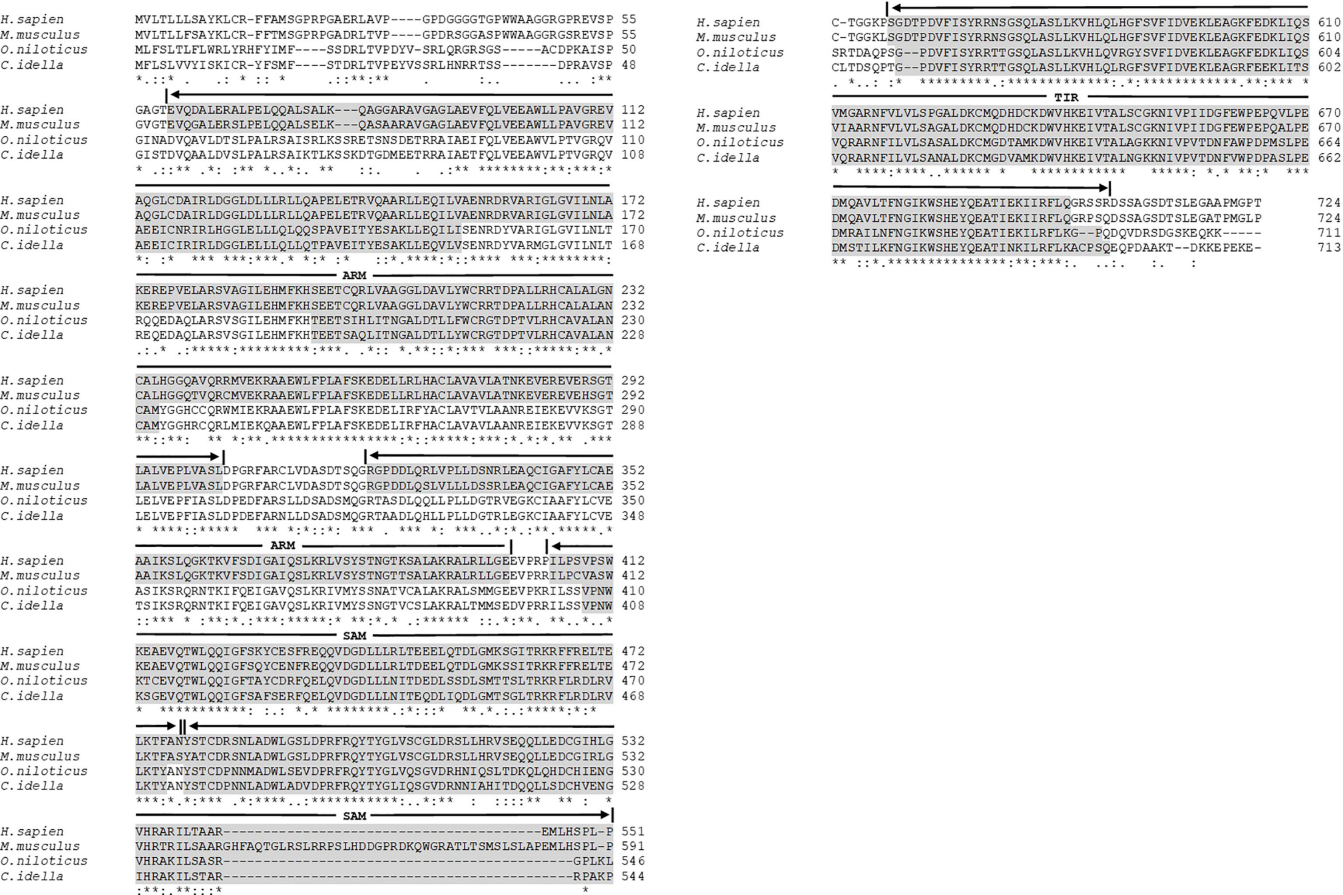
Multiple alignments of SARM1 amino acid sequences from *Oreochromis niloticus*, *Homo sapiens*, *Mus musculus*, and *Ctenopharyngodon idella*. The accession numbers of the amino acid sequences are provided in **Supplementary Table 2**. The dashes in the sequences indicate gaps introduced to maximize alignment. Identical (*), similar (.), and highly conserved (): residues identified by the ClustalW program are indicated. Five domains are labeled and shaded in grey. ARM, Armadillo/beta-catenin-like repeats; SAM, Sterile alpha motif; and TIR, Toll - interleukin 1 - receptor.

The characterized domains of OnSARM1 are shown in **Figure 3**. OnSARM1, similar to other SARM1s, contains two N-terminal armadillo/beta-catenin-like repeats (ARM) domains, two central sterile alpha motifs (SAM), and a C-terminal TIR domain.

**Figure 4:**
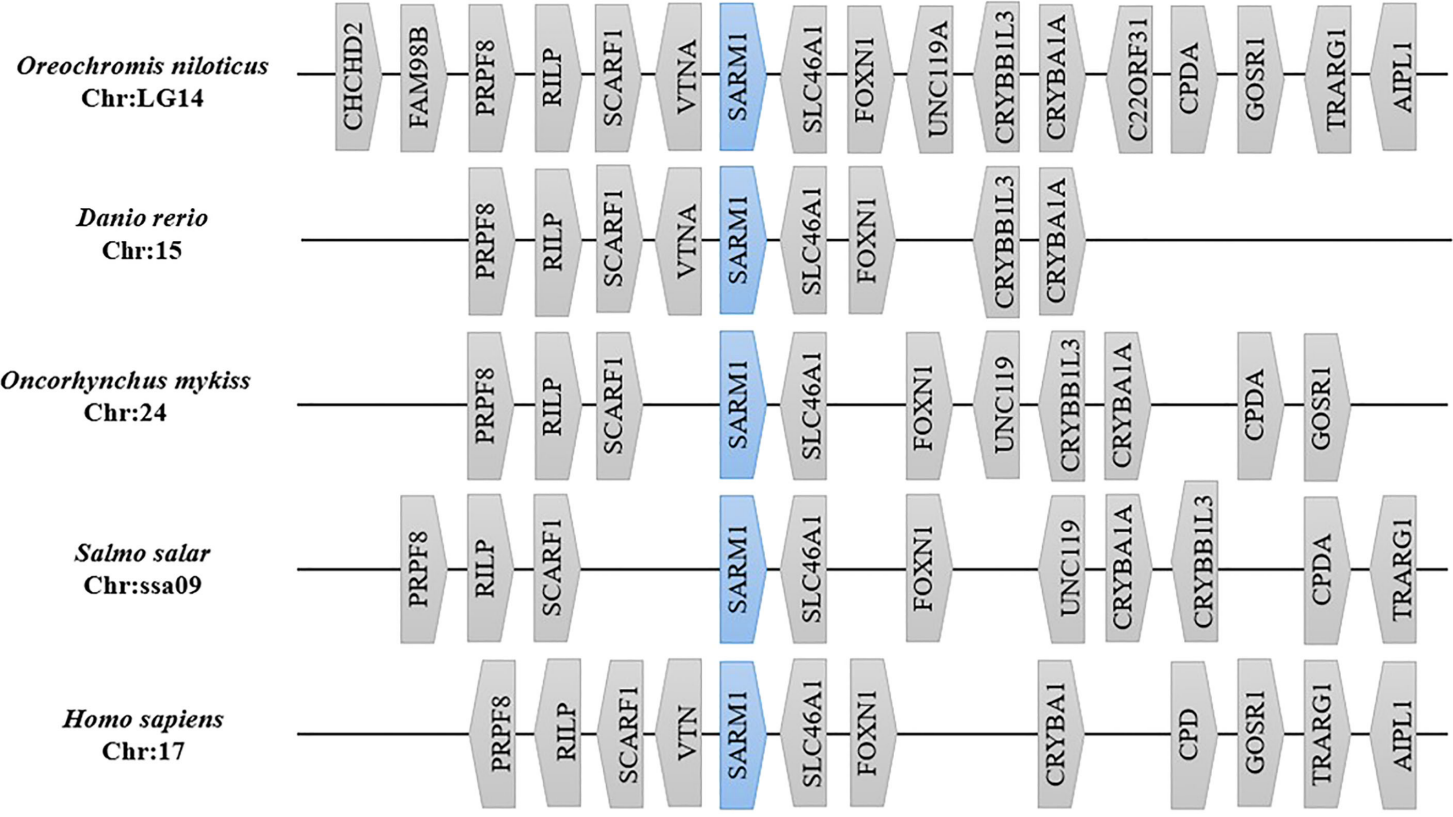
Gene synteny analysis of segments containing SARM1 from different species. Gene order on the chromosome of each species was collected using the Genomicus database (Version 100.01). Gene distance was not drawn to scale. Gene names are given as follows: CHCHD2, Coiled-coil-helix-coiled-coil-helix domain containing 2; FAM98B, Family With Sequence Similarity 98 Member B; PRPF8, Pre-mRNA-processing-splicing factor 8; RILP, Rab-interacting lysosomal protein; SCARF1, Scavenger Receptor Class F Member 1; VTNA, vitronectin a; SARM1, Sterile alpha and TIR motif-containing protein 1; SLC46A1, Solute Carrier Family 46 Member 1; FOXN1, Forkhead box protein N1; UNC119A, Protein unc-119 homolog A; CRYBB1L3, crystallin beta B1d; CRYBA1A, Beta A1-crystalline; C22ORF31, chromosome 22, open reading frame 31; CPDA, 3’,5’-cyclic adenosine monophosphate phosphodiesterase; GOSR1, Golgi SNAP receptor complex member 1; TRARG1, Trafficking regulator of GLUT4 1; AIPL1, Aryl-hydrocarbon-interacting protein-like 1; UNC119, Uncoordinated-119; VTN, Vitronectin; CRYBA1, Crystallin Beta A1; and CPD, Carboxypeptidase D.

**Figure 4** presents a comparison of the genome segments containing *SARM1* from various fishes and humans. The genes flanking *SARM1* were conserved among vertebrates analyzed. Specifically, the gene block containing *OnSARM1* was similar to that of *Danio rerio*, with SLC46A1 (Solute Carrier Family 46 Member 1) and FOXN1 (Forkhead box protein N1) located downstream, and VTNA (vitronectin a), SCARF1 (Scavenger Receptor Class F Member 1), RILP (Rab-interacting lysosomal protein), and PRPF8 (Pre-mRNA-processing-splicing factor 8) located upstream of *SARM1*.

### Expression of *OnSARM1* in tissues of healthy fish

**Figure 5:**
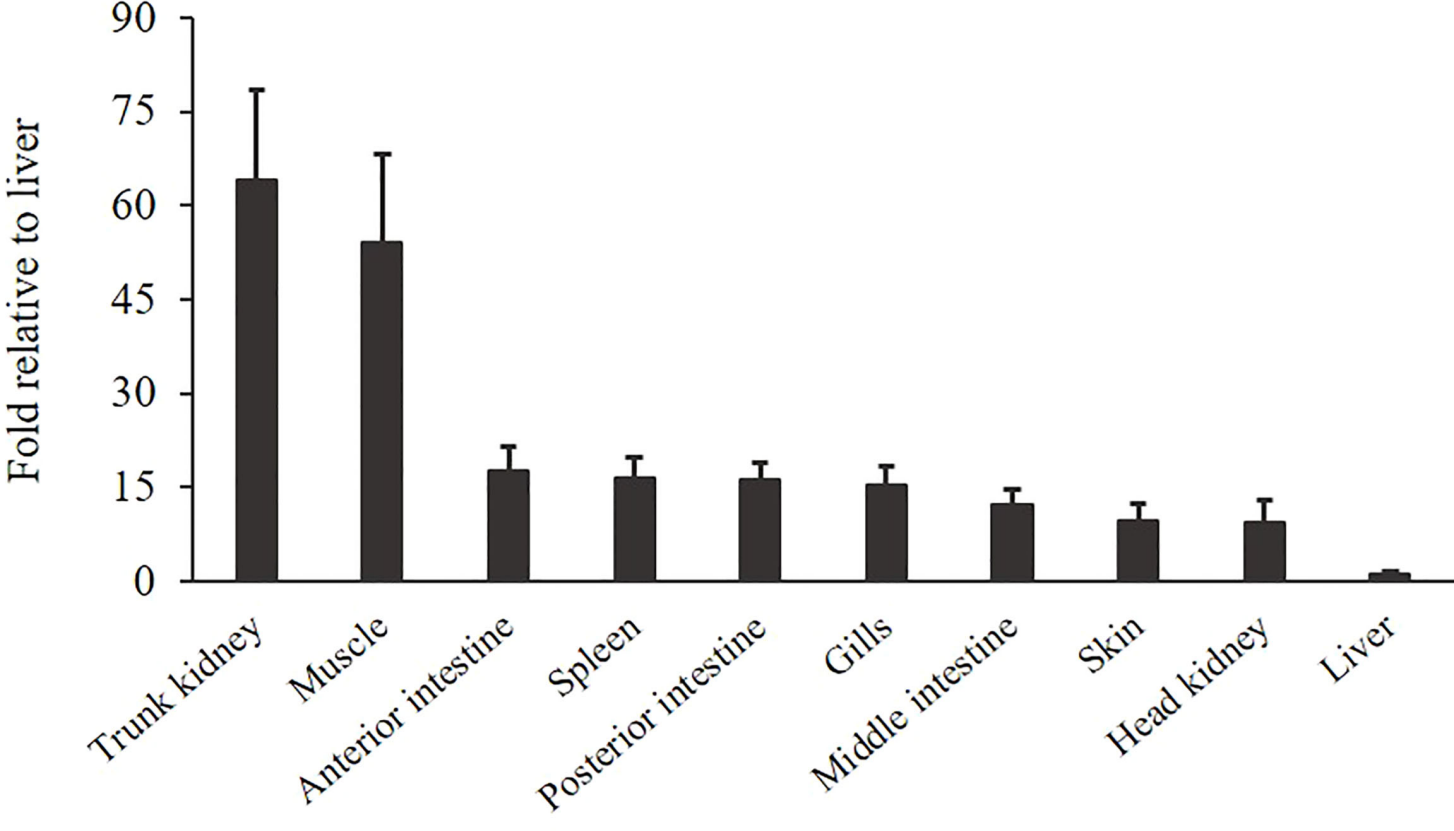
Gene expression of *OnSARM1* in tissues from healthy tilapia. The expressions of *OnSARM1* in the trunk kidney, muscle, anterior intestine, spleen, posterior intestine, gills, middle intestine, skin, head kidney, and liver were analyzed using real-time PCR and normalized to the expression of *EF-1α*. Data are expressed as mean + SD (n = 4) and presented as fold change relative to the liver.

The gene expression of *OnSARM1* in the tissues from healthy Nile tilapia is shown in **Figure 5**. The expression of *OnSARM1* was highly expressed in the trunk kidney (64.2-fold) followed by the muscle (54.1-fold), when compared with the liver that exhibited the lowest expression (set as 1), and it was expressed moderately in the intestine, spleen, gills, skin, and head kidney.

### Cellular localization of OnSARM1

**Figure 6:**
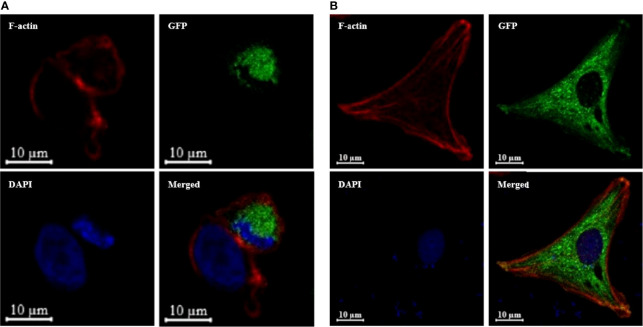
OnSARM1 is preferentially located in the intracellular region. **(A)** HEK 293 and **(B)** THK cells were transfected with pTurbo-OnSARM1-GFP expression plasmid (green). The cells were fixed 48 h after transfection, incubated with Alexa Fluor 546 phalloidin to visualize F-actin Cytoskeleton (red), stained with VECTASHIELD Antifade Mounting Medium containing DAPI (blue), and observed under a confocal microscope (Zeiss LSM 880 with Airyscan).

An expression plasmid (pTurbo-OnSARM1-GFP), which encoded for OnSARM1 tagged with GFP at the C-terminus of the TIR domain, was constructed to study the cellular location of OnSARM1. Successful expression of the fusion protein was confirmed by the Western blotting of lysates from pTurbo-OnSARM1-GFP transfected HEK 293 and THK cells (**Supplementary Figure 2**). The molecular weight of the OnSARM1-GFP fusion protein was approximately 106 kDa (26 kDa Turbo-GFP plus 80 kDa for SARM1), while the pTurbo-GFP vector transfected cells produced a 26 kDa GFP protein. OnSARM1-GFP fusion protein was detected only in the cytoplasm of HEK 293 and THK cells (**Figure 6**).

### Gene expression analysis in Nile tilapia after being challenged with *S. agalactiae* and *A. hydrophila*

**Figure 7:**
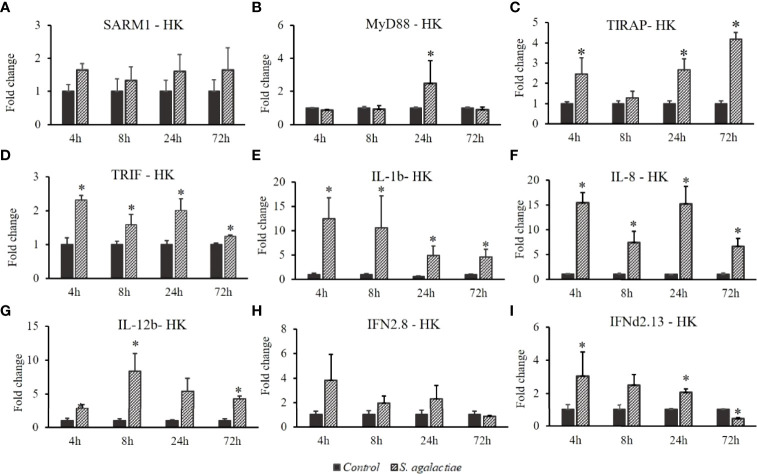
Gene expression of **(A)** *SARM1*, **(B)** *MyD88*, **(C)** *TIRAP*, **(D)** *TRIF*, **(E)** *IL-1β*, **(F)** *IL-8*, **(G)** *IL-12b*, ***(*H*)*** *IFNd2.8*, and **(I)** *IFNd2.13* in the head kidney from *Oreochromis niloticus* challenged with *Streptococcus agalactiae*. Expression of the indicated genes was normalized to the expression level of *EF-1α* and expressed as fold change relative to the control group. The values are shown as mean + SD (n = 3). Significant differences from the control group at each time point are indicated by asterisks (P < 0.05, unpaired sample t-test).

The expression profiles of *OnSARM1*, *OnMyD88*, *OnTIRAP*, *OnTRIF*, *IL-1β*, *IL-8*, *IL-12b*, *IFNd2.8* and *IFNd2.13* were determined in the head kidney after a challenge with Gram-positive bacteria (*S. agalactiae*) (**Figure 7**). The expression levels of *OnSARM1* (**Figure 7A**) and *IFNd2.8* (**Figure 7H**) were not significantly altered at any of the time points examined, while the transcript levels of *OnTIRAP* (**Figure 7C**) and *OnTRIF* (**Figure 7D**) increased significantly in the head kidney at 3 and 4 time points after being challenged, and *OnMyD88* was upregulated at 24 h (**Figure 7B**). Significant upregulation of *IL-1β* (**Figure 7E**) and *IL-8* (**Figure 7F**) was observed at all of the time points examined post *S. agalactiae* infection. Elevated gene expression of *IL-12b* and *IFNd2.13* was noticed at 8 h and 72 h, and 4 h and 24 h, respectively in the head kidney tissue from the *S. agalactiae* challenged fish.

**Figure 8:**
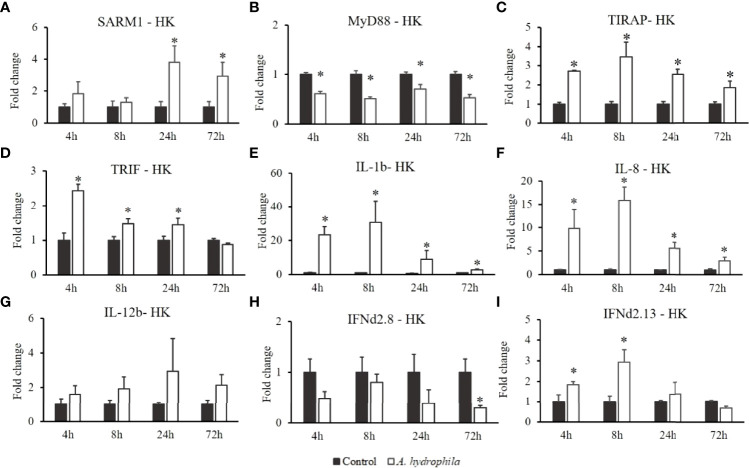
Gene expression of **(A)** *SARM1*, **(B)** *MyD88*, **(C)** *TIRAP*, **(D)** *TRIF*, **(E)** *IL-1β*, **(F)** *IL-8*, **(G)** *IL-12b*, **(H)** *IFNd2.8*, and **(I)** *IFNd2.13* in the head kidney from *Oreochromis niloticus* challenged with *Aeromonas hydrophila*. Expression of the indicated genes was normalized to the expression level of *EF-1α* and expressed as fold change relative to the control group. The values are shown as mean + SD (n = 3). Significant differences from the control group at each time point are indicated by asterisks (P < 0.05, unpaired sample t-test).

In the fish challenged with Gram-negative bacteria (*A. hydrophila*), the transcript levels of *OnSARM1* (**Figure 8A**) were promoted at 24 h and 72 h, while *OnMyD88* was downregulated in the head kidney after the challenge injection (**Figure 7B**). The expressions of *OnTIRAP* (**Figure 8C**) and *OnTRIF* (**Figure 8D**) were upregulated at 4 and 3 time points post *A. hydrophila* infection. Similarly, the expression of *IL-1β* (**Figure 8E**) and *IL-8* (**Figure 8F**) reached the highest level at 8 h, and decreased gradually at later time points. The expression of *IL-12b* (**Figure 8G**) and *IFNd2.8* (**Figure 8H**) remained almost at the basal level, but *IFNd2.13* (**Figure 8I**) was upregulated at 4 h and 8 h in the head kidney post *A. hydrophila* infection.

### Interplay of OnMyD88, OnTRIF, OnTIRAP and OnSARM1 and their influence in NF-κB activation

**Figure 9:**
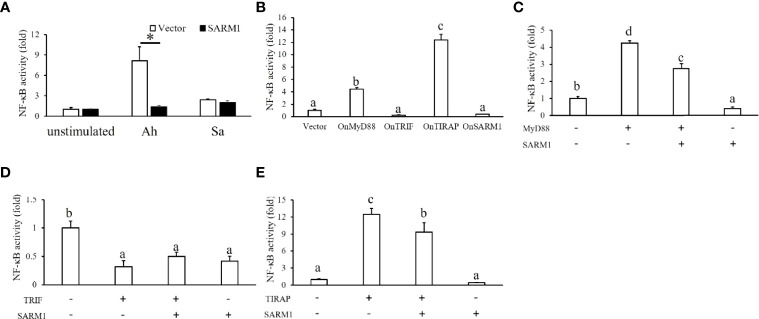
Luciferase assay on the activation of a NF-κB-luc reporter by OnMyD88, OnTRIF, OnTIRAP, and OnSARM1 in THK cells. **(A)** THK cells were transfected with the pNF-κB-Luc reporter with an empty vector or OnSARM1-3×FLAG expression plasmids. Forty-eight hours post transfection, the cells were treated with lysates from *Aeromonas hydrophila* (Ah, 10 μg bacterial protein/ml culture medium) or *Streptococcus agalactiae* (Sa, 10 μg bacterial protein/ml culture medium) for 16 h; this was followed by luciferase assay analysis (n = 3). **(B)** Activity of the NF-κB-luc reporter was determined in the THK cells transfected with the expressing plasmids of *OnMyD88*, *OnTRIF*, *OnTIRAP*, and *OnSARM1* (n = 3). **(C–E)** THK cells were co-transfected with the NF-κB-luc reporter together with *OnMyD88*, *OnTRIF*, *OnTIRAP*, and *OnSARM1* expressing plasmids alone or in a combination of two, and the corresponding empty vector was used as a control. Forty-eight hours post transfection, the cells were harvested to detect luciferase activity (n = 3). The values are shown as mean *+* SD. Significant differences between the OnSARM1 or vector transfected group [**(A)**, unpaired sample t-test] or between the experimental groups [**(B–E)**, one-way ANOVA and *Tukey’s post-hoc* test] are indicated by asterisks or different letters (P < 0.05), respectively.

To further study the regulatory role of OnSARM1 in immune cells, we adopted a cell line derived from tilapia head kidney with characteristics of melanomacrophages (26). Enhanced NF-κB promoter activity was seen in the empty vector transfected THK cells after treatment of the lysates from *A. hydrophila* (8.17 ± 2.05 fold) and *S. agalactiae* (2.42 ± 0.16 fold). Interestingly, *A. hydrophila*-mediated induction of NF-κB promoter activity was significantly suppressed in the OnSARM1 overexpressed THK cells (1.93 ± 0.21 fold) (**Figure 9A**). Although insignificant, this inhibitory effect was also seen in the *S. agalactiae* treated THK cells that overexpressed OnSARM1 (1.99 ± 0.31 fold).

In order to investigate the interaction among OnSARM1 and other TLR adaptors, the corresponding expressing plasmids were transfected into THK cells in combination with NF-κB reporter plasmids. As shown in **Figure 9B**, overexpression of OnMyD88 and OnTIRAP alone, but not OnTRIF and OnSARM1, activated NF-κB activity in THK cells, but the co-expression of OnSARM1 with three other adaptors, namely OnMyD88, OnTRIF and OnTIRAP, impaired the activation of the NF-κB reporter (**Figures 9C**–**E**).

### Co-localization of OnSARM1 and other TLR adaptors

**Figure 10:**
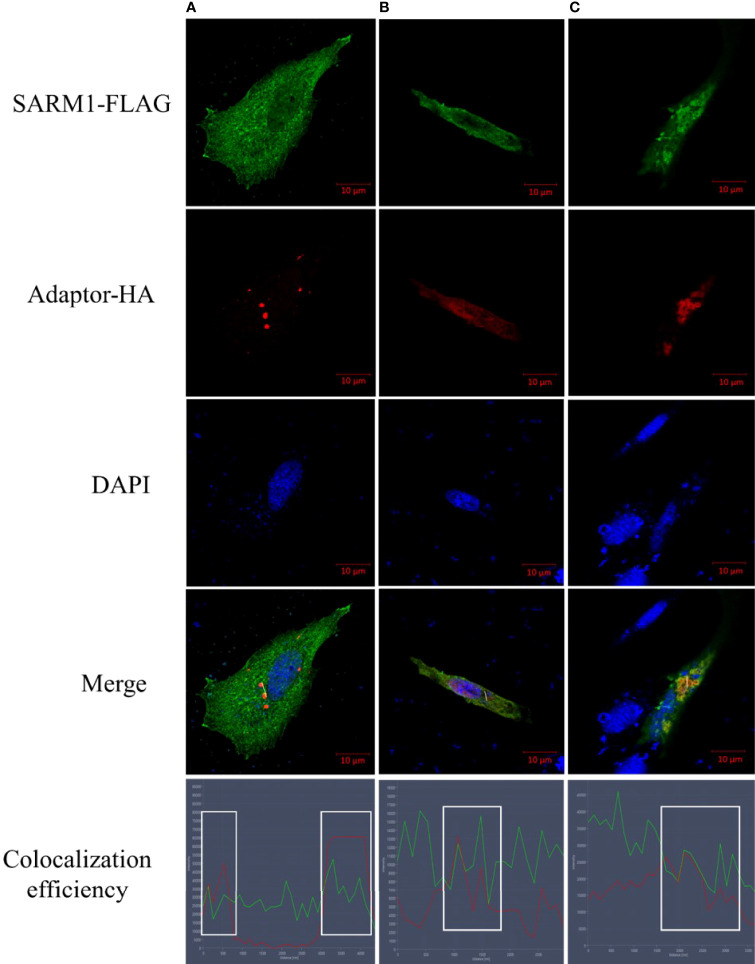
Colocalization of OnSARM1 with other TLR adaptors. THK cells were co-transfected with OnSARM1-3×FLAG and HA-tagged **(A)** OnTRIF, **(B)** OnTIRAP or **(C)** OnMyD88 expression plasmids, respectively. The cells were fixed 48 h after transfection, followed by staining with anti-FLAG or anti-HA antibodies. Alexa Flour™ 488 goat anti-mouse IgG and Alexa Flour™ 568 goat Anti-rabbit IgG were used to visualize FLAG tag (green) or HA tag (red), respectively. Cells were then stained with VECTASHIELD Antifade Mounting Medium containing DAPI to visualize nucleus (blue). Images were captured using confocal microscopy (Zeiss LSM 880 with Airyscan). Colocalization efficiency was quantified by observing the overlap of fluorescence intensity peaks along the white line by line scan analysis profiles (ZEISS ZEN 2.6 (blue edition) software).

Studies have shown that SARM1 could physically interact with other TLR adaptors by using co-immunoprecipitation and Western blotting (20), but whether OnSARM1 could co-localize with other TLR adaptors still needs to be determined. To this end, THK cells were transfected with OnSARM1-3×FLAG together with HA-tagged OnTRIF, OnTIRAP or OnMyD88 expression plasmids respectively, and the cells were stained with corresponding antibodies 48 h post-transfection. As shown in **Figure 10**, OnSARM1 were detected not only scattered in the cytoplasmic region but also formed in discrete foci in the cytosolic region that were found to be partially co-localized with OnTRIF (**Figure 10A**) or OnMyD88 (**Figure 10C**), which are present as small speckle-like condensed granules in plasmid transfected THK cells. OnTIRAP was found to be more evenly distributed in cytosol and co-localized with OnSARM1 (**Figure 10B**). Colocalization of OnSARM1 with the three TLR adaptors was visible as yellow coloring in the merged images and was further confirmed by the correspondence of the peak fluorescence intensities of the indicated proteins in the line scan graphs (**Figure 10**, bottom row).

### OnSARM1 inhibits proinflammatory cytokine and antimicrobial peptide genes in THK cells post *A. hydrophila* lysate stimulation

**Figure 11:**
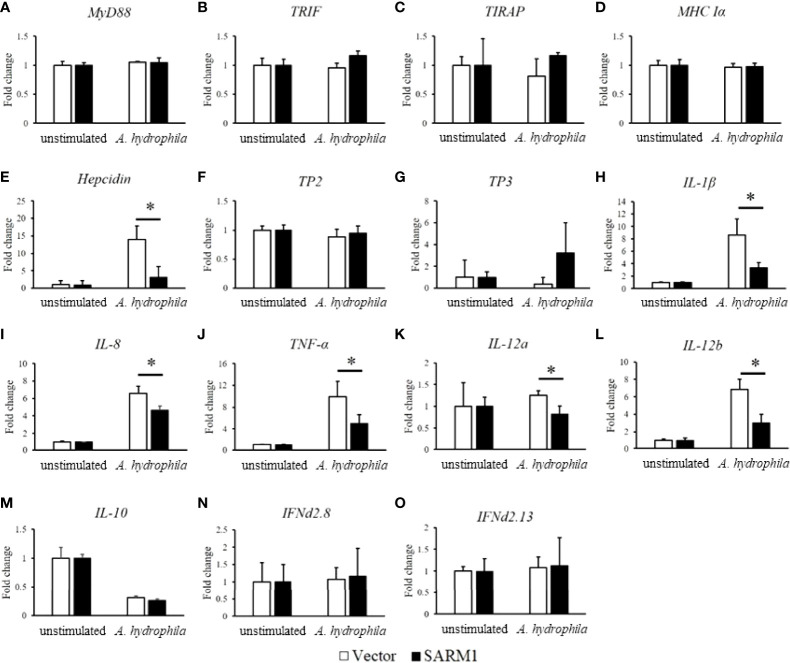
OnSARM1 inhibits mRNA expression of immune genes **(A)** *MyD88*, **(B)** *TRIF*, **(C)** *TIRAP*, **(D)** *MHC* Ia, **(E)** *Hepicidin*, **(F)** *TP2*, **(G)** *TP3*, **(H)** *IL-1b*, **(I)** *IL-8*, **(J)** *TNF-a*, **(K)** *IL-12a*, **(L)** *IL-12b*, **(M)** *IL-10*, **(N)** *IFNd2.8*, **(O)** *IFNd2.13* in *Aeromonas hydrophila* lysate stimulated THK cells. THK cells were transfected with empty vector or OnSARM1-3×FLAG expression plasmids. Forty-eight hours post transfection, the cells were treated with lysates from *A. hydrophila* (10 µg bacterial protein/ml culture medium) for 4 h; this was followed by gene expression analysis (n=3). The values are shown as mean + SD. Significant differences from the vector group are indicated by asterisks (P < 0.05, unpaired sample t-test).

We next examined the effect of OnSARM1 on anti-*A. hydrophila* dependent gene induction in more detail by comparing the mRNA expression level of immune genes in empty vector or OnSARM1 expressing plasmid transfected THK cells after stimulation with lysate from *A. hydrophila*. As shown in **Figure 11**, expression of *MyD88*, *TRIF* and *TIRAP* (TLR adaptors), *tilapia piscidin 2* (*TP2*) and *TP3* (antimicrobial peptide), *major histocompatibility complex class Ia* (*MHC Ia*), *interferon* (*IFN*)*d2.8* and *IFNd2.13*, and *IL-10* remained at a similar level compared to the empty vector transfected group. In contrast, *hepcidin* (antimicrobial peptide) was upregulated in the empty vector group to nearly 14-fold (13.94 ± 3.89 fold) after stimulation with lysate from *A. hydrophila*, but was suppressed in the cells overexpressed OnSARM1 (3.05 ± 3.15 fold) (**Figure 11E**). The suppressive effects were further exemplified by evidence of the downregulation of proinflammatory cytokine genes such as *IL-1β* (8.64 ± 2.56 fold versus 3.34 ± 0.84 fold), *IL-8* (6.60 ± 0.80 fold versus 4.64 ± 0.47 fold), *TNF-α* (9.88 ± 2.81 fold versus 5.04 ± 1.56 fold), *IL-12a* (1.25 ± 0.11 fold versus 0.82 ± 0.19 fold), *IL-12b* (6.85 ± 1.19 fold versus 3.03 ± 0.96 fold) between the vector or OnSARM1 plasmid transfected THK cells after stimulation (**Figure 11**).

## Discussion

Among the five known TLR adaptors, MyD88, TIRAP, TRIF, TRAM (absent in the fish genomes), and SARM1, SARM1 was the last to be identified. It is a pro-apoptotic protein in mammals that functions as a suppressor of TLR signaling (14, 30). However, in teleosts, SARM1’s function and role remain unclear. In this study, the CDS of *OnSARM1* was cloned, and bioinformatic analyses suggested that the cloned gene was a homolog of SARM1 in Nile tilapia.

The results of the sequence alignment showed that OnSARM1 contained two N-terminal ARM domains, two central SAM motifs, and a C-terminal TIR domain, which is identical to the homologs from grass carp, mouse, and humans (13). In mammals, the first 27 amino acids at the N-terminal of SARM have been shown to be hydrophobic and polybasic, and to act as a mitochondria-targeting signal sequence for associating SARM to the mitochondria (30). From the sequence alignment, we noticed that four amino acids “GPRP” out of the first 27 amino acids of SARM1 were missing in fish, but the specificity of the mitochondria-targeting ability of the arginine residue at the 14th position was conserved between fish and mammalian SARM1 (**Figure 3**), suggesting that the subcellular localization of fish SARM1 is a conserved feature. Indeed, CiSARM1 was identified as specifically localized to mitochondria in the CIK cell (13).

The basal expression patterns of *SARM1* were found to differ among various species. For example, *CiSARM1* was expressed at high levels in the foregut, skin, and eye and at a low level in the midgut (13), while the homologous gene was expressed at high levels in the heart and gills in *Litopenaeus vannamei* and at a low level in the hemocyte (18). In mandarin fish (*Siniperca chuatsi*), *SARM1* was detected in all of the tissues examined and was found to be highly expressed in the trunk kidney, skin, intestine, and pyloric caecum (20). In this study, the expression of *OnSARM1* was found to be at a high level in the trunk kidney and muscle, and its lowest expression was in the liver.

The head kidney is an important lymphoid tissue in the immune system of fish (31). So far, only a few investigations have studied *SARM1* expression modulation in fish challenged with bacteria. We therefore investigated the expression of *OnSARM1* in the head kidney after challenging Nile tilapia with two pathogenic bacteria, *A. hydrophila* and *S. agalactiae*. The expression of *OnMyD88* was not always modulated in the same way as *OnTIRAP*, and we also noticed that the expression of *IL-1β* was modulated in a similar way to that of *OnTIRAP* and *OnTRIF*, but was not correlated with the regulation of *OnMyD88* in the head kidney from bacterial challenged fish. IL-1β is a critical pro-inflammatory cytokine that is essential in response to infection and is thought to be induced mainly through the TLR/MyD88/NF-κB signaling pathway in monocytes/macrophages (32). However, a recent study has elegantly demonstrated that both MyD88 and TRIF contribute to the priming of *IL-1β* expression and pro–IL-1β protein production in murine myeloid DCs after monophosphoryl lipid A (MLA, a derivative of LPS) stimulation (33). Additionally, TIRAP, but not MyD88, was shown to play an essential role in phosphoinositide 3-kinase (PI3K) activity and NF-κB activation upon diacylated lipoprotein stimulation (34). Therefore, a plausible explanation of the induction of *OnIL-1β* (and other proinflammatory cytokines) while *OnMyD88* was downregulated (or not upregulated) in the head kidney from *A. hydrophila* and *S. agalactiae* injected fish is that it is likely to be triggered by the TRIF- and/or TIRAP- dependent pathways.

IL-8, a CXC chemokine, is produced by macrophages and other cell types to mainly attract and activate neutrophils (35), and is regulated through the TLR/MyD88/NF-κB signaling pathway (36, 37). Studies have revealed that the activation of MyD88-dependent signaling pathways will promote the expression of *IL-1β* and *IL-12b* (*p40*) in murine peritoneal macrophages and RAW264.7 cells (38, 39) and *IL-8* in porcine alveolar macrophages (37). Herein, the transcript level of *OnIL-8* was sharply induced by bacterial infection/stimulation (for THK cells), indicating that it is a significant factor in host defense in the head kidney and in the pathogenesis of bacterial infection in Nile tilapia.

IL-12 is a heterodimeric cytokine composed of the p35 (known as IL-12a) and p40 (known as IL-12b) subunits, which are important for stimulating the development of T helper 1 cells and cell-mediated immunity against pathogens (40). IL-12 is secreted by a range of cell types, including macrophages, neutrophils, and dendritic cells. Various microbial specific signatures (e.g., LPS, CpG, and peptidoglycan) are recognized by TLRs (41), leading to induction of the expression of IL-12b in the MyD88/TIRAP-dependent pathway (42), which is (partially) regulated by NF-κB (p50/p65 and p50/c-Rel) complexes in phagocytes (40). The expression of *OnIL-12b* was greatly enhanced in the head kidney after *S. agalactiae* infection (moderately upregulated by *A. hydrophila* infection) and in the THK cells by the treatment of *A. hydrophila* lysate, possibly due to the activated MyD88/TIRAP-NF-κB-IL-12b cascade. Interestingly, *OnIL-12b* expression was inhibited in the OnSARM1 overexpressed THK cells; such a suppressive effect was also seen in adult brains from *SARM1* knockdown transgenic mice (43).

Type I IFNs can be induced by viruses and bacteria. The main producing cells are macrophages, dendritic cells, and other hemopoietic cells (44). Stimulation of murine bone-marrow-derived DCs with *Lactobacillus acidophilus* (45) or murine macrophages with lipoteichoic acid (LTA) from *Staphylococcus aureus* (46) induced IFN-β or IFN-α production, respectively, in a TLR2-dependent pathway. Additionally, unmethylated CpG DNA, which is expressed approximately 20 times more frequently in bacterial than vertebrate DNA (47), was recognized by TLR9 (48). TRIF is a signaling adaptor, which can activate IRF3, NF-κB, and AP1 and induce type I IFN and cytokines (49, 50). In this study, the expression of *OnSARM1* and *OnTRIF* was mutually fluctuated in the immune tissues after *A. hydrophila* was administered, signifying that *OnSARM1* and *OnTRIF* may act together in the antimicrobial immune responses but are regulated in the opposite way. For example, when the fish were infected with *A. hydrophila*, the transcript levels of *OnTRIF* and *OnIFNd2.13* were upregulated in the head kidney at early time points (4 h and 8 h), while the *OnSARM1* expression was unaltered; however, *OnSARM1* upregulated at later time points (24 h and 72 h), whereas the transcript levels of *OnTRIF* and *OnIFNd2.13* reverted to a level similar to that of the control group (**Figure 8**). This suggests that OnSARM1 plays a role as a negative regulator in the TRIF-mediated pathway to prevent excessive inflammation (14).

Bacterial infection (or lysates) causes the delivery of ligands, such as LTA, peptidoglycan, flagellin, single-stranded (ss) RNA, double-stranded (ds) RNA and CpG DNA, to the host, which activates multiple TLRs, such as TLR1-3, TLR5, TLR7-9, TLR21, and teleost-specific TLR19 (51) and TLR22 (52). Additionally, our recent research revealed that the transcript levels of Nile tilapia *TLR18* (27) and *TLR25* (28) were elevated in the head kidney during bacterial infection and that these two fish-specific TLRs can physically interact with both MyD88 and TRIF, adding the possibility that they may recognize (unknown) bacteria-derived ligands and launch the production of proinflammatory cytokines and type I IFNs after activation.

Subsequent to an investigation conducted on human SARM (14), studies have also suggested that fish SARM1 serves as a negative regulator in antiviral responses (13, 20). However, the function of SARM1 in antibacterial responses at the cellular level remains unexplored in teleost. Here, the NF-κB reporter was activated in the *A. hydrophila* and *S. agalactiae* treated THK cells, and for the first time we showed that this inductive effect was inhibited in the OnSARM1 overexpressed THK cells (**Figure 9**). Moreover, gene expression of proinflammatory cytokines and hepcidin was evoked in THK cells by the treatment of lysate derived from *A. hydrophila*, but was suppressed when OnSARM1 was overexpressed (**Figure 11**), supporting the notion that SARM1 acts as a negative regulator in antibacterial responses in fish.

Carty et al. demonstrated that human MyD88- and TIRAP-mediated NF-κB activation in HEK 293 cells was unaffected by SARM expression, while TRIF-induced NF-κB activity was inhibited by SARM1 in a dose-dependent manner (14). By contrast, co-expression of mandarin fish MyD88, TRIF, or TIRAP with SARM1 in HEK 293 cells resulted in impaired NF-κB promoter activity mediated by the former three adaptors (20). Overexpression of OnMyD88 and OnTIRAP can induce the activity of the NF-κB-luc reporter in HEK 293 cells (21, 27) and THK cells, indicating that the function of these TLR adaptors in fish is conserved, as found in mammals. Similar to the findings in mandarin fish, overexpression of OnSARM1 suppressed OnMyD88*-* and OnTIRAP-mediated NF-κB promoter activity, presumably through the physical interaction of the BB-loop of the SARM1 TIR domain and the TIR domain from MyD88 and TIRAP (53). Additionally, Carlsson et al. showed that both SARM and MyD88 localize to the mitochondria (53), which is similar to the results from the confocal microscopy analysis in the present study, in which OnSARM1 colocalized with OnTRIF, OnMyD88 or OnTIRAP in small speckle-like condensed granules in the cytoplasm. Although enhanced NF-κB activity was seen in OnTRIF overexpressed HEK 293 cells (27), this was not the case when transfecting the same expression plasmid (for overexpressing OnTRIF) in the THK cells, and the presence of OnSARM1 had no influence on the activity of the NF-κB-luc reporter. The underlying mechanisms for the discrepancy in the OnTRIF-mediated NF-κB activity between human and fish cells need to be identified and may be of interest for further research.

## Conclusions

In conclusion, *OnSARM1* was cloned, identified, and characterized in Nile tilapia. The expression of *OnSARM1* and other TLR adaptors was regulated in the head kidney after microbial stimulation. OnSARM1 colocalized with OnMyD88, OnTIRAP or OnTRIF in the cytoplasm of cells. Moreover, OnSARM1 alone was unable to activate the NF-κB reporter, but can suppress NF-κB promoter activity mediated by OnMyD88, OnTRIF and OnTIRAP in THK cells. Finally, OnSARM1 is likely to serve as a negative regulator in the NF-κB pathway during antibacterial responses, and inhibit the gene expression of proinflammatory cytokines and hepcidin in melanomacrophage-like cells.

## Data availability statement

The original contributions presented in the study are included in the article/**Supplementary Material**, further inquiries can be directed to the corresponding author.

## Ethics statement

The animal study was reviewed and approved by NTOU Institutional Animal Care and Use Committee (approval number: 109042).

## Author contributions

P-TL designed and supervised the study. PN, NT, H-YH, J-YL, EW, and AW conducted the study and data analysis. PN and NT wrote the first version of the manuscript. All authors contributed to the article and approved the submitted version.

## Funding

This work was funded by the Ministry of Science and Technology (MOST) under grant agreement no. MOST 110-2313-B-019-010-MY3.

## Conflict of interest

The authors declare that the research was conducted in the absence of any commercial or financial relationships that could be construed as a potential conflict of interest.

## Publisher’s note

All claims expressed in this article are solely those of the authors and do not necessarily represent those of their affiliated organizations, or those of the publisher, the editors and the reviewers. Any product that may be evaluated in this article, or claim that may be made by its manufacturer, is not guaranteed or endorsed by the publisher.
