# Supplementary material for: Sterile alpha and TIR motif-containing protein 1 is a negative regulator in the anti-bacterial immune responses in nile tilapia (Oreochromis niloticus)
Source: Front Immunol. 2022 Jul 19;13:940877. doi: 10.3389/fimmu.2022.940877 (PMC9344004; doi:10.3389/fimmu.2022.940877)
Supplement: Supplementary file 1 [file DataSheet_1.docx]

Supplementary Material

00010gcatccacatcgatcacattgtttgtgccggtagATGCTTTTCTCCCTGACGCTTTTCTTG
 H **1** 23332  H  R  S  H  C  L  C  R  -\  **M  L  F  S  L  T  L  F  L** 
00620TGGAGGCTCTACCGACATTTCTACATCATGTTCAGCTCTGACCGGCTCACAGTCCCGGAT

**10**00**W  R  L  Y  R  H  F  Y  I  M  F  S  S  D  R  L  T  V  P  D**
01220TACGTCAGCAGGCTGCAGAGAGGGAGGAGCGGCTCCGCATGCGACCCCAAGGCCATCTCT
 0**30**00**Y  V  S  R  L  Q  R  G  R  S  G  S  A  C  D  P  K  A  I  S**
01820CCGGGGATCAACGCCGACGTCCAGGCGGTTTTGGACACGTCACTTCCCGCCTTGCGCTCC
 0**50**00**P  G  I  N  A  D  V  Q  A  V  L  D  T  S  L  P  A  L  R  S**
02420GCCATCAGTAGGCTGAAGTCATCCAGGGAGACCTCGAATTCAGATGAGACCCGCAGGGCT
 0**70**00**A  I  S  R  L  K  S  S  R  E  T  S  N  S  D  E  T  R  R  A**
03020ATCGCGGAGATCTTCCAGCTGGTGGAGGAGGCCTGGGTTTTACCTACCGTGGGTCGTCAA
 0**90**00**I  A  E  I  F  Q  L  V  E  E  A  W  V  L  P  T  V  G  R  Q**
03620GTGGCCGAGGAGATCTGCAACAGGATCCGGCTGCATGGAGGCCTGGAGCTGCTGCTTCAG
 **110**00**V  A  E  E  I  C  N  R  I  R  L  H  G  G  L  E  L  L  L  Q**
04220CTCCAGCAGTCGCCTGCTGTGGAGATCACTTACGAGTCCGCAAAACTGCTGGAGCAGATA
0**130**0**L  Q  Q  S  P  A  V  E  I  T  Y  E  S  A  K  L  L  E  Q  I**
04820CTGATCTCAGAGAacagggattacGTGGCTCGCATAGGTCTGGGGGTCATCCTCAACCTG
0**150**0**L  I  S  E  N  R  D  Y  V  A  R  I  G  L  G  V  I  L  N  L**
05420ACTCGTCAGCAGGAAGATGCTCAGCTGGCTCGGAGTGTCTCGGGCATCCTCGAGCACATG
 **170**00**T  R  Q  Q  E  D  A  Q  L  A  R  S  V  S  G  I  L  E  H  M**
06020TTCAAACACACAGAGGAGACCTCCATCCACCTCATCACCAACGGCGCCCTGGACACCCTC
 **190**00**F  K  H  T  E  E  T  S  I  H  L  I  T  N  G  A  L  D  T  L**
06620CTCTTCTGGTGCCGAGGTACGGACCCCACTGTTCTGCGCCACTGTGCCGTGGCACTGGCT
0**210**0**L  F  W  C  R  G  T  D  P  T  V  L  R  H  C  A  V  A  L  A** 
07220AACTGTGCGATGTATGGAGGCCACTGCTGCCAGCGCTGGATGATCGAGAAACGGGCAGCT
 **230**0**N  C  A  M  Y  G  G  H  C  C  Q  R  W  M  I  E  K  R  A  A**
07820GAGTGGCTCTTCCCACTCGCCTTTTCCAAAGAGGATGAACTCATTCGTTTCTATGCCTGC
 **250**00**E  W  L  F  P  L  A  F  S  K  E  D  E  L  I  R  F  Y  A  C**
08420TTGGCTGTAACTGTGTTAGCTGCAAACAGAGAAATTGAGAAAGAGGTGGTGAAATCAGGG
 **270**00**L  A  V  T  V  L  A  A  N  R  E  I  E  K  E  V  V  K  S  G**
09020ACCTTGGAGCTAGTGGAGCCGTTCATCGCATCTCTGGATCCAGAAGACTTTGCCCGCAGT
 **290**00**T  L  E  L  V  E  P  F  I  A  S  L  D  P  E  D  F  A  R  S**
09620TTACTGGACAGTGCAGACAGCATGCAGGGAAGGACCGCATCTGACCTGCAGCAACTTTTG
 **310**00**L  L  D  S  A  D  S  M  Q  G  R  T  A  S  D  L  Q  Q  L  L**
10220CCGTTACTGGATGGCACGAGAGTGGAGGGAAAGTGCATTGCAGCCTTTTACCTGTGTGTT
 **330**00**P  L  L  D  G  T  R  V  E  G  K  C  I  A  A  F  Y  L  C  V**
10820GAGGCCAGCATCAAGTCCCGTCAGCGCAACACTAAGATATTTCAAGAGATTGGTGCTGTG
 **350**00**E  A  S  I  K  S  R  Q  R  N  T  K  I  F  Q  E  I  G  A  V**
11420CAGAGCCTCAAAAGAATCGTCATGTACTCCAGCAATGCCACAGTCTGTGCCCTAGCTAAG
 **370**00**Q  S  L  K  R  I  V  M  Y  S  S  N  A  T  V  C  A  L  A  K**
12020CGGGCGCTGAGCATGATGGGAGAAGAAGTACCGAAACGAATCCTGTCAAGCGTGCCCAAC
 **390**00**R  A  L  S  M  M  G  E  E  V  P  K  R  I  L  S  S  V  P  N**
12620TGGAAAACCTGTGAGGTGCAGACGTGGCTGCAGCAGATCGGCTTCACCGCTTACTGTGAC
0**410**0**W  K  T  C  E  V  Q  T  W  L  Q  Q  I  G  F  T  A  Y  C  D**
13220CGTTTTCAGGAACTTCAGGTGGATGGAGACCTCCTTCTGAACATCACAGATGAAGATCTG
0**430**0**R  F  Q  E  L  Q  V  D  G  D  L  L  L  N  I  T  D  E  D  L**

13820AGCTCTGATTTGAGCATGACTACCAGCCTTACCCGCAAGAGGTTTTTGAGAGACTTGCGT
0**450**0**S  S  D  L  S  M  T  T  S  L  T  R  K  R  F  L  R  D  L  R**
14420GTGCTGAAGACTTACGCCAATTACTCCACATGTGACCCCAACAACATGGCCGACTGGTTA
0**470**0**V  L  K  T  Y  A  N  Y  S  T  C  D  P  N  N  M  A  D  W  L** 
15020AGCGAGGTGGACCCCCGTTTTCGTCAGTACACCTATGGCCTAGTCCAGTCAGGTGTGGAC
0**490**0**S  E  V  D  P  R  F  R  Q  Y  T  Y  G  L  V  Q  S  G  V  D**
15620CGTCACAACATCCAGAGCCTGACCGATAAGCAGCTCCAGCACGACTGCCACATTGAGAAT
0**510**0**R  H  N  I  Q  S  L  T  D  K  Q  L  Q  H  D  C  H  I  E  N**
16220GGCGTCCACAGAGCAAAGATACTGTCTGCCAGCCGTGGGCCCTTAAAACTGAGCCGCACA
0**530**0**G  V  H  R  A  K  I  L  S  A  S  R  G  P  L  K  L  S  R  T**
16820GATGCACAGCCTTCAGGGCCTGACGTTTTTATCAGCTACAGACGAACCACGGGCTCCCAG
 **550**00**D  A  Q  P  S  G  P  D  V  F  I  S  Y  R  R  T  T  G  S  Q**
17420CTGGCCAGCCTATTGAAGGTGCACCTGCAGGTTCGAGGATACAGCGTCTTCATAGATGTG
0**570**0**L  A  S  L  L  K  V  H  L  Q  V  R  G  Y  S  V  F  I  D  V**
18020GAGAAGCTGGAGGCCGGTAAATTTGAGGACAAACTGATTCAGAGTGTGCAGAGGGCACGT
0**590**0**E  K  L  E  A  G  K  F  E  D  K  L  I  Q  S  V  Q  R  A  R**
18620AACTTCATCCTGGTCCTGTCTGCCAGTGCACTCGACAAGTGTATGGGAGACACAGCCATG
0**610**0**N  F  I  L  V  L  S  A  S  A  L  D  K  C  M  G  D  T  A  M**
19220AAGGACTGGGTGCATAAGGAGATAGTCACGGCCCTGGCTGGTAAGAAGAACATAGTTCCT
0**630**0**K  D  W  V  H  K  E  I  V  T  A  L  A  G  K  K  N  I  V  P**
19820GTCACGGATAACTTTGCATGGCCTGACCCCATGTCCCTGCCAGAAGACATGAGAGCCATT
0**650**0**V  T  D  N  F  A  W  P  D  P  M  S  L  P  E  D  M  R  A  I**
20420CTCAACTTCAACGGCATCAAGTGGTCCCACGAATATCAGGAGGCTACGATCGAGAAGATC
0**670**0**L  N  F  N  G  I  K  W  S  H  E  Y  Q  E  A  T  I  E  K  I**
21020CTGCGCTTTCTGAAAGGACCCCAAGACCAAGTCGACCGCTCAGACGGCTCCAAAGAGCAG
0**690**0**L  R  F  L  K  G  P  Q  D  Q  V  D  R  S  D  G  S  K  E  Q**
21620AAGAAATAAactgagataaacacagtccgatttcagctataataa
 **710**00**K  K  -**T

Supplementary Fig. 1. Nucleotide and deduced amino acid sequence of OnSARM1 and numbered on the left of each row. The start codon (ATG) and stop codon (TAA) were boxed. Untranslated regions (UTR) were shown in lowercase. In the deduced amino acid sequence, two ARM repeats domains (111 - 151 aa, 193 - 233 aa) were in bold and underlined, two SAM domains (407 - 474 aa, 477 - 546 aa) were double underlined, and TIR domain (555 - 696aa) was shaded in grey.


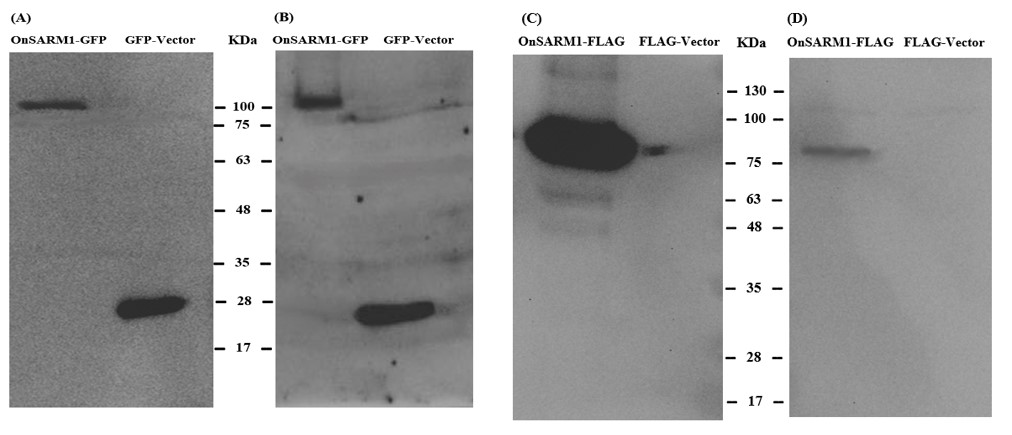


Supplementary Fig. 2. Western blot analysis of OnSARM1-GFP (A & B) and OnSARM1-3×FLAG (C & D) fusion proteins. HEK 293 (A & C) and THK (B & D) cells were transfected with pTurbo-OnSARM1-GFP expression plasmid (left lane) or pTurbo-GFP-N empty vector (right lane, as control in both panels). HEK 293 (C) and THK (D) cells were transfected with pCMV-OnSARM1-3×FLAG-FL plasmid (left lane) or pCMV-3×FLAG-14 vector (right lane, as control in both panels). 48 h after transfection, cells were washed and lysed. Lysates were separated by a gradient SDS-PAGE gel, then transferred to a PVDF membrane, and probed with anti-TurboGFP or anti-FLAG antibodies, respectively.


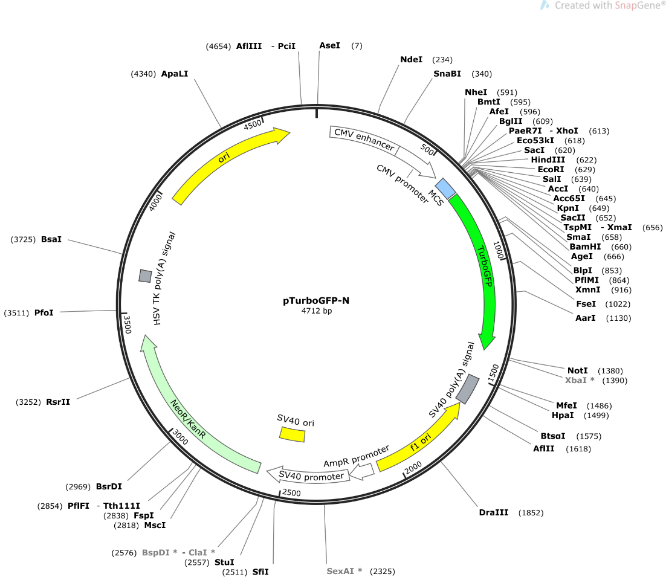

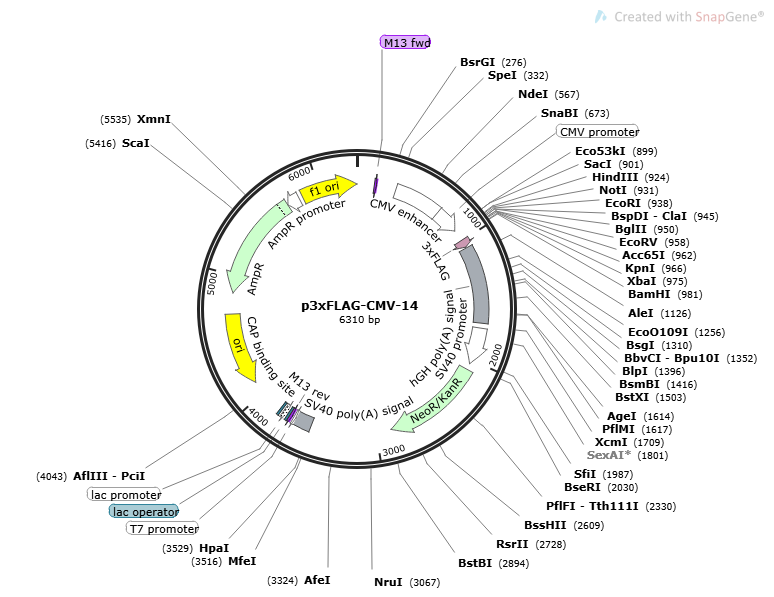


**A**

**B**

Supplementary Fig. 3. Expression vector maps of p3XFLAG-CMV™-14 (A) and pTurbo-GFP-N (B)

Supplementary Table 1. Primers used in this study

| Primer | Sequence (5' ⭢ 3') | Application |
| --- | --- | --- |
| EF-1α-QF | GAGAGCTTCAACGCTCAGGT | Real-time PCR |
| EF-1α-QR | CACGGCGAAACGACCGAGG | Real-time PCR |
| qOnSARM1-F | GGACTGGGTGCATAAGGAG | Real-time PCR |
| qOnSARM1-R | GATATTCGTGGGACCACTTGAT | Real-time PCR |
| qOnTRIF-F | GCCTACAACTTCAGCAGAAAG | Real-time PCR |
| qOnTRIF-R | TCCTGTCCCATGGTTGGC | Real-time PCR |
| qOnMyD88-F | GTGAACTCATCGAGAAGAGGT | Real-time PCR |
| qOnMyD88-R | GTTTACTTCGAGCTCCAGGA | Real-time PCR |
| qTIRAP-F | GACGGTACTCCAGTACCTG | Real-time PCR |
| qTIRAP-R | TTCTTGTAAGCTGTCTGCTCC | Real-time PCR |
| qIL-1β-F | CAGTGAAGACCGCAAAGTGC | Real-time PCR |
| qIL-1β-R | TATCCGTCACCTCCTCCAG | Real-time PCR |
| qIL-8-F | TCGCCACCTGTGAAGGCAT | Real-time PCR |
| qIL-8-R | TCCTTTTCAGTGTGGCAATGAT | Real-time PCR |
| qIL-12a-F | GAAATTCTTCCTTCAGTGAGAGT | Real-time PCR |
| qIL-12a-R | TGTGGAAAGCAGTTCTCTGTG | Real-time PCR |
| qIL-12b-F | CAACAGTGACAATCAAATAATTAATAT | Real-time PCR |
| qIL-12b-R | CGTTATGTTTGTTCACTGTGCA | Real-time PCR |
| qTNFα-F | GAACACTGGCGACAAAACAGA | Real-time PCR |
| qTNFα-R | TTGAGTCGCTGCCTTCTAGA | Real-time PCR |
| qIFNd2.8-F | AAGAGCTTCACTCCTGTATTG | Real-time PCR |
| qIFNd2.8-R | GCTTCAGCACTGTAACCATTT | Real-time PCR |
| qIFNd2.13-F | GAGCTTCGCTCCTGTATTGG | Real-time PCR |
| qIFNd2.13-R | GGCTTCAGCACTGTAACCATTT | Real-time PCR |
| qTP2-QF | ATGAAGTGTGCTGCAGTATTTCTTATGCTGTCC | Real-time PCR |
| qTP2-QR | CTAGTCAAAATTAAGTCGACGAGGGGT | Real-time PCR |
| qTP3-QF | ATGAAGTGCACCATGCTGTTCCTTGTGCTGTCGATGGTT | Real-time PCR |
| qTP3-QR | CTAGTTAAAAGCAGCCCTTTCCC | Real-time PCR |
| qHepcidin-F | GTCCCTGTTGTTGAAGAGCA | Real-time PCR |
| qHepcidin-R | TCCATTGTGCAGCATCTTCCA | Real-time PCR |
| qIL10-F | GTCGAAGACTCCTTTAAGAGC | Real-time PCR |
| qIL10-R | CAGGAGAAGTATTTCCTACACT | Real-time PCR |
| qMHC Ia-F | AGAGCCGAGATGGTCTGGA | Real-time PCR |
| qMHC Ia-R | AGAGAGCTGAAACACACATGTGT | Real-time PCR |
| FL-OnSARM1-F | GCATCCACATCGATCACATTG | Cloning of OnSARM1 |
| FL-OnSARM1-R | TATTATAGCTGAAATCGGACTGTG | Cloning of OnSARM1 |
| GFP-*Nhe*I- OnSARM1-F | CTAGCTAGCTAGATGCTTTTCTCCCTGACG | Construction of pTurbo- OnSARM1-GFP plasmid |
| GFP-*Hind*III-OnSARM1-R | CCCAAGCTTTTTCTTCTGCTCTTTGGAGC | Construction of pTurbo- OnSARM1-GFP plasmid |
| FLAG-*Hind*IIIOnSARM1-FL-F | CCCAAGCTTTAGATGCTTTTCTCCCTGACG | Construction of pCMV- OnSARM1-3×FLAG |
| FLAG-*Xba*IOnSARM1-FL-R | CTAGTCTAGATTTCTTCTGCTCTTTGGAGC | Construction of pCMV- OnSARM1-3×FLAG |
| GFP-colony-F | CGCAAATGGGCGGTAGGCGTG | Colony PCR on pTurbo-GFP-N vector |
| GFP-colony-R | CTCGAACTCCACGCCGTT | Colony PCR on pTurbo-GFP-N vector |
| CMVF | CGTGTACGGTGGGAGGTCTA | Colony PCR on pCMV-3×FLAG-14 |
| pCMV-Flag-14R | CACCCGGGATCACTACTTGT | Colony PCR on pCMV-3×FLAG-14 |

Supplementary Table 2. Accession numbers of selected SARM1 sequences.

| Species | GenBank accession number |
| --- | --- |
| African clawed frog (*Xenopus laevis*) | XP_018104264.1 |
| Arabian camel (*Camelus dromedarius*) | XP_031324220.1 |
| Arabian camel (*Camelus dromedarius*) | XP_031324220.1 |
| Astatotilapia burtoni(*Haplochromis burtoni*) | XP_005915969.1 |
| Atlantic herring (*Clupea harengus*) | XP_012680673.2 |
| Cat (*Oryctolagus cuniculus*) | XP_008269261.1 |
| Catfish (*Ictalurus punctatus*) | XP_017346861.1 |
| Chicken (*Gallus gallus*) | XP_415814.4 |
| Eastern river bream (*Astatotilapia calliptera*) | XP_026047784.1 |
| Flier cichlid (*Archocentrus centrarchus*) | XP_030600581.1 |
| Giant grouper (*Epinephelus lanceolatus*) | XP_033487626 |
| Guppy (*Poecilia reticulata*) | XP_008423818.1 |
| Human (*Homo sapiens*) | NP_055892.2 |
| Indian medeka (*Oryzias melastigma*) | XP_024133639.1 |
| Japanese flounder (*Paralichthys olivaceus*) | XP_019943913.1 |
| Japanese medeka (*Oryzias latipes*) | XP_004075485.1 |
| Japanese quail (*Coturnix japonica*) | XP_015736009.1 |
| Monterrey platyfish (*Xiphophorus couchianus*) | XP_027855055.1 |
| Mouse (*Mus musculus*) | NP_001161993.1 |
| Mummichog (*Fundulus heteroclitus*) | XP_012732823.2 |
| Ocellaris clownfish (*Amphiprion ocellaris*) | XP_023150335.1 |
| Pig (*Cavia porcellus*) | XP_003469614.1 |
| Prehistoric Monster Fish (*Thalassophryne amazonica*) | XP_034025248.1 |
| Pygmy chimpanzee (*Pan paniscus*) | XP_003812708.1 |
| Rabbit (*Oryctolagus cuniculus*) | XP_008269261.1 |
| Seahorse (*Hippocampus comes*) | XP_019741446.1 |
| Southern platyfish (*Xiphophorus maculatus*) | XP_005801097.1 |
| Spotted gar (*Lepisosteus oculatus*) | XP_015223043.1 |
| Striped catfish (*Pangasianodon hypophthalmus*) | XP_026790376.1 |
| Prehistoric Monster Fish (*Thalassophryne amazonica*) | XP_034025248.1 |
| Tilapia (*Oreochromis niloticus*) | XP_003458672.1 |
| Tropical clawed frog (*Xenopus tropicalis*) | XP_031752536.1 |
| yellow perch (*Perca flavescens*) | XP_028429728.1 |
| Zebra mbuna (*Maylandia zebra*) | XP_004543863.1 |
| zebu cattle (*Bos indicus*) | XP_019837654.1 |

Supplementary Table 3. Amino acid identity and similarity of SARM1s between Oreochromis niloticus and other selected vertebrates.

|  | *Oreochromis niloticus* | |
| --- | --- | --- |
|  | Identity (%) | Similarity (%) |
| *Lates calcarifer* | 91.7 | 95.9 |
| *Epinephelus lanceolatus* | 89.5 | 94.2 |
| *Salmo salar* | 83.3 | 89.5 |
| *Danio rerio* | 82.0 | 90.2 |
| *Ictalurus punctatus* | 77.6 | 87.3 |
| *Xenopus tropicalis* | 62.0 | 76.6 |
| *Gallus gallus* | 61.1 | 77.0 |
| *Homo sapiens* | 59.6 | 74.2 |
| *Cavia porcellus* | 59.0 | 74.2 |
| *Mus musculus* | 56.9 | 70.6 |
